# Fifteenth Annual Meeting of the American Dental Convention

**Published:** 1870-01

**Authors:** J. H. Smith

**Affiliations:** Rec. Secretary.


### ARTICLE VI.

### *Fifteenth Annual Meeting of the American Dental Convention.*

The association of Dentists organized in August, 1855, under the name of the AMERICAN DENTAL CONVENTION held its *fifteenth annual meeting*, at Tyler's Hall, New Haven, Conn., on Thursday October 21st, 1869.

The President DR. J. M. CROWELL of Brooklyn, N. Y. called the meeting to order at eleven o'clock A. M.

The Secretary being absent Dr. Samuel Mallet of New Haven, was appointed Secretary pro-tem.

The regular order of business was proceeded with which with discussions on various professional subjects, and the election of officers for ensuing year occupied the time of the morning session.

The election of officers resulted in the choice for President of Dr. J. G. Ambler of New York, for Vice-President Dr. Samuel Mallett of New Haven, for Recording Secretary and Treasurer Dr. J. H. Smith of New Haven, for Corresponding Secretary Dr. J. S. Latimer of New York.

The retiring President made a short address in which he urged upon the members of the profession to use their utmost endeavors to discontinue the use of rubber or vulcanized dental plates which are working so much injury to the health of the public, and to pay more attention to the improvements of a better class of work.

He stated he had of late been studying the merits of Porcelain Base, and was pleased with the improvements made in the style of work; also that the cheapness and ease with which rubber was made, had the tendency to drive out from mechanical dentistry the better class of dentists, but that he hoped now, since it was found that rubber was injurious as a base for artificial dentures, that it would bring back the more skillful dentists to a higher standard in the mechanical department; while we have improved greatly in the operative department, there has been a falling off in artificial work.

The incoming President, Dr. J. G. Ambler of New York, on taking the chair alluded to the remarks which had just been made and stated that instances were numerous in his practice where vulcanized rubber plates injured the health of the wearer. Considered gold and platina far preferable in every respect; thought we ought to perfect instead of cheapen artificial dentures.

Dr. Ambler in his address gave a very pleasant reminiscence of the convention from its birth up to the present time, showing the good which it had done in bringing the dentist that was no operator at all, up to a standard that our best operators of the present day are not ashamed to acknowledge as well worthy the confidence of the public &c., &c.,

Dr. Preterre of New York was opposed to the use of rubber; he considered the collodion far better for cheap work. He exhibited a number of specimens of collodion and rose pearl, but for durability and nice work he considered gold plates or Allen's continuous gum or platina as the best that could be used.

Dr. Mallett of New York said that he had met with the same difficulties that the preceeding gentleman had with rubber, that it had been a curse to the profession, the cheapness of the work and the ease with which it was made had lowered the dignity of the profession, bringing a class of people into our ranks that were not worthy the name of dentist, who extracted thousands of teeth that might otherwise have been saved. Its use had done a great deal toward introducing the adominable custom of extracting sound teeth, which he regarded as one of the most horrible practices. Nevertheless rubber has been a perfect boon to thousands who could not afford a gold plate, and its utility hinges upon the question whether or no it was a proper article to put in the mouth. He was in favor of gold plate.

Dr. J. H. Smith said that in his practice for full sets he preferred the continuous gum or platina plate, had discarded rubber entirely. For partial pieces, used gold or iridium; had in some iustauces made full sets of iridium plate, baking the Allen siliceous compound the same as platina plate; it made a much stiffer plate and required a little more care in striking up than platina, for it was not as yielding, and when strength was required he preferred it; had had good success in the continuous gum work far less breakage than with rubber. While he made rubber he preferred continuous gum work, and as a general thing the breaking of these sets was from letting them fall while washing over a marble or china basin, thinks its the strongest work made; while in the mouth he said the weight was often made an objection to the continuous gum by dentists using rubber, but he had found no trouble in that way when a good fit was made, had less trouble in getting a good fitting plate than he used to have when he was using rubber, had frequently made the continuous gum work for patients that were wearing rubber plates and to use their own expression, "They fit better and feel lighter than my rubber ones did." Properly made he believed the continuous gum on platina or iridium plates was, all things considered, the very best that he knew of as yet for artificial dentures, &c.

After a lively discussion of this and various other subjects, it was on motion resolved that when we adjourn it be to meet in the city of New York, on the third Tuesday in September, 1870.

The President appointed Dr. W. B. Hind of Brooklyn; Dr. B. T. Whitney of Buffalo; Dr. I. J. Wetherbee of Boston; Dr. L. S. Straw of Newburg and Dr. John Allen of New York as Executive Committee, and Drs. W. H. Atkinson, F. H. Clark, N. W. Kingsley of New York; Dr. Marvin of Brooklyn, Dr. W. H. Morgan of Nashville, Dr. W. T. Arrington of Memphis Tenn., Dr. F. J. S. Gorgas of Baltimore, Dr. M. S. Dean of Chicago, Dr. L. W. Rogers and C. A. Foster of Utica, Dr. A. Westcott of Syracruse, Dr. C. W. Harvey of Buffalo, Dr. F. French of Rochester, Drs. Jas. Truman and A. Tees of Philadelphia, Dr. H. L. Ambler of Cleveland Ohio, Dr. F. Searl of Springfield Mass., and Dr. Riggs of Hartford Ct., as Essayists.

On motion adjourned.

J. H. Smith, *Rec. Secretary.*

